# A cohort description and analysis of the effect of gabapentin on idiopathic cough

**DOI:** 10.1186/1745-9974-8-9

**Published:** 2012-11-01

**Authors:** Charlotte Van de Kerkhove, Pieter C Goeminne, Pascal Van Bleyenbergh, Lieven J Dupont

**Affiliations:** 1 Department of Internal Medicine, UZ Leuven, Leuven, Belgium; 2 Department of Respiratory Disease, UZ Leuven, Herestraat 49, 3000, Leuven, Belgium

## Abstract

**Background:**

Chronic idiopathic cough (known as cough hypersensitivity syndrome) is defined by cough in the absence of an identifiable cause. Gabapentin has been suggested as a treatment but evidence is scarce. The aim of our study was to describe the clinical features of patients with unexplained chronic cough and to investigate the effect of gabapentin (600 mg twice a day for a minimal duration of 4 weeks) in reducing cough symptoms.

**Methods:**

A patient cohort analysis was performed. Patients were retrieved using a query in our medical database for the words ‘cough’ and ‘gabapentin’ in 2011. Patients without a clear etiology of cough despite having performed a stepwise diagnostic approach, were included. Medical records of these patients were analyzed. A telephonic survey was performed and patients were asked to retrospectivally rate their cough when they attended the outpatient clinic. They were then asked to rate their cough after treatment with gabapentin. A scale from one to ten was used to score cough severity. They were also questioned about the triggers inducing cough. To evaluate the cough severity score, the results were correlated with questions of the Leicester Cough Questionnaire.

**Results:**

We recruited 51 patients (87% female) with a mean age of onset of 47 years (± 14 y) and an average cough duration of 48 months. The most frequently reported cough triggers included change of temperature (57%), talking (49%) and odours (45%). In 67% of patients, the urge to cough was located in the throat area. Thirty-five patients effectively took the prescribed gabapentin. The average improvement in cough score was 2.8/10 (p<0.0001). Of the 35 patients, 20 achieved improvement of their cough symptoms. Responders had a higher pre-treatment cough severity score (p=0.02) and were more likely to have a history of pre-cough airway infection (p=0.04). Current cough severity score negatively correlated with the Leicester Cough Questionnaire scores (p=0.05).

**Conclusion:**

Chronic idiopathic cough were predominantly middle-aged women, frequently reporting various cough triggers. We also demonstrated that gabapentin can significantly improve cough in these patients. Responders tend to have higher pre-treatment severity scores and have a history of an airway infection.

## Background

Chronic cough often remains a diagnostic and therapeutic challenge. It is associated with a significant impaired quality of life and health care cost. Current guidelines suggest the use of both diagnostic tests and empirical treatment trials in its management [1]. The most common conditions associated with chronic cough are gastro-esophageal reflux disease, asthma syndromes and upper airway disorders such as rhinitis or rhinosinusitis [2]. A final diagnosis of chronic idiopathic cough is made when there is no identifiable cause [3,4]. A universal characteristic of these patients is an abnormally sensitive cough reflex. Therefore the term ‘cough hypersensitivity syndrome’ was recently introduced [5]. The greatest challenge in these patients is downregulating this cough hypersensitivity. Fortunately, several novel mechanisms have been identified, which may lead to the identification of targets that could lead to new effective antitussives [6]. As pathophysiological mechanisms are thought to be similar between chronic cough and neuropathic pain, gabapentin was recently tried as a potential treatment for chronic idiopathic cough [7,8]. In a randomized, double-blind, placebo-controlled trial, Ryan et al. show that gabapentin is a well-tolerated therapy that significantly improves cough-specific quality of life, frequency of cough and severity [8].

The aim of this article is to describe the clinical characteristics of the patient cohort with idiopathic cough seen at our chronic cough outpatient clinic and recent empirical experience of efficacy of treatment with gabapentin in this population.

## Methods

Patients were recruited using a query in our medical database. The keywords ‘*cough*’ and ‘*gabapentin*’ and an outpatient clinic visit in 2011 were the selection criteria. All hits were analyzed for presence of chronic idiopathic cough. We defined idiopathic cough as a cough that lasted for more than eight weeks in the absence of any abnormality in the clinical examination, chest radiograph, CT sinuses, lung function, negative histamine provocation test, differential cell count of induced sputum and no pathological reflux during a 24 hours pH/impedance monitoring. In addition to the diagnostic work-up, all patients underwent empirical treatment trials with proton pump inhibitors (≥ 6 weeks of omeprazole 40mg twice daily), nasal decongestants, (≥ 6 weeks fluticasone 100μg twice daily or equivalent) and inhaled steroids ((≥ 6 weeks fluticasone 250μg twice daily or equivalent).

Individual files were then analyzed for patient characteristics looking at gender, age of onset, prior upper airway infections, duration of cough symptoms, smoking habits, cough triggers and response to trial therapies given.

Patients who did not improve under the previous therapies were put on gabapentin. A minimum therapy duration of at least four weeks was suggested using an initial dose of 300 mg for four days, increasing with 300 mg each four days until a maintenance dose was reached of 600 mg twice daily. Patients needed a treatment period of four weeks or more to be included into the analysis.

All patients were seen at the outpatient clinic one to two months later to evaluate therapy. Patients were asked about the nature and severity of their cough by means by a pre-set list of questions. We also contacted all patients using a telephone survey. They were asked to retrospectively score their cough severity on a scale of ten before they attended the outpatient clinic. A score of zero was equal to no cough and a score of ten was the worst cough possible. Subsequently they were asked to score their present cough severity (after gabapentin treatment).

To validate this cough severity scale, we also added four questions of the Leicester Cough Questionnaire [9], using the Dutch version [10], to correlate these scores with the cough severity scale. The Leicester Cough Questionnaire questions asked were: *In the last 2 weeks, my cough has interfered with my job, or other daily tasks; In the last 2 weeks, has your cough disturbed your sleep?; In the last 2 weeks, how many times a day have you had coughing bouts?; In the last 2 weeks, my cough has interrupted conversation or telephone call.* Each question is scored from one to seven and a lower score indicates higher impact of cough on quality of life.

Approval was obtained from the local ethical committee of UZ Leuven, Belgium and patients were asked by telephone if the data could be used for anonymous analysis.

Results were expressed as mean with standard deviation in case of normal distribution or as median with interquartile range for non-normal data. Paired *t*-test or Wilcoxon signed-rank test was used according to distribution of the data as were unpaired *t*-test and Mann–Whitney *U* test. Correlations were analyzed using a Pearson analysis for parametric data or Spearman’s rank analysis for non-parametric data. P-values reached significance if lower than 0.05 and two-tailed testing was used. Analysis was performed using GraphPad Prism 4.01.

## Results

### Patient characteristics

We collected data from 51 patients. Forty-one (80%) were female, with a mean age of onset of cough of 47 years (SD ± 14 years). Median duration of chronic cough before outpatient visit was 48 months (IQR 2 – 192) and 28% had a history of an airway infection. Patients almost universally complained of a dry, non-productive cough, with irritation and discomfort localized in the throat (67%) or in the chest area (33%), leading to paroxysms of coughing. Diurnal variation showed daytime predominance in 69% of the population and nocturnal predominance in 12%.

Strong associations were also seen when triggers of cough were investigated. Changes in temperature, mainly transition to cold outside, was a trigger in 57% of patients. Other triggers such as talking (41%), strong odors (31%), physical effort (20%) and eating (29%) were also common (Table 1). Patients pointed out that even a minimal trigger induced their cough which is indicative of their increased cough reflex sensitivity.

**Table 1:** Triggers of cough: Table showing total number of patients (and percentage) suffering coughing bouts if exposed to the specific trigger

**Cough triggers**	**N (%)**
Temperature change	29 (57%)
Talking	21 (41%)
Strong odors	16 (31%)
Smoke	19 (37%)
Meals	15 (29%)
Exercise	10 (20%)
Stress	8 (16%)
Dust	5 (10%)
None	4 (8%)

### Effect of gapapentin

**Figure 1:**
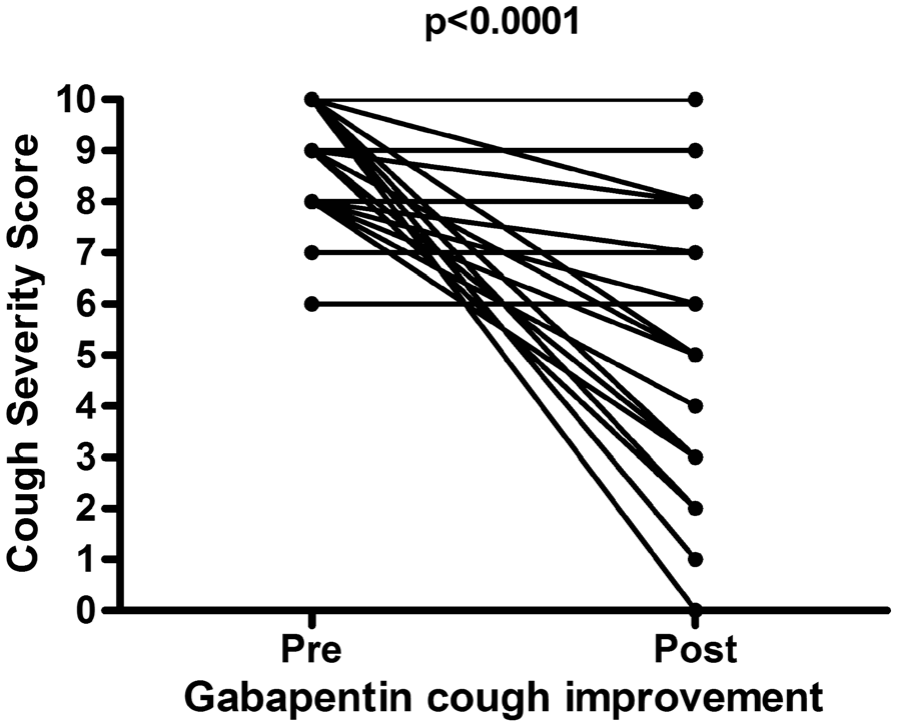
Cough Severity score before and after the start of gabapentin: A significant improvement is seen of the cough severity score after start of gabapentin (p<0.0001; 95% Confidence interval 1.7–3.9).

Our retrospective analysis showed that 43 patients effectively took gabapentin. Eight subjects discontinued during treatment due to adverse effects (fatigue (5) and dizziness (3)). Another eight subject did not start the treatment because of a fear of side-effects. Of a total of 35 patients who completed their treatment with gabapentin, a mean reduction in cough severity score of 2.8 was seen (p<0.0001) (Figure 1). Subanalysis showed that an improvement in cough score was seen in 20 (57%) of these patients with complete remission (cough score 0) in two patients. No patient reported an increase in cough symptoms during their treatment.

**Figure 2:**
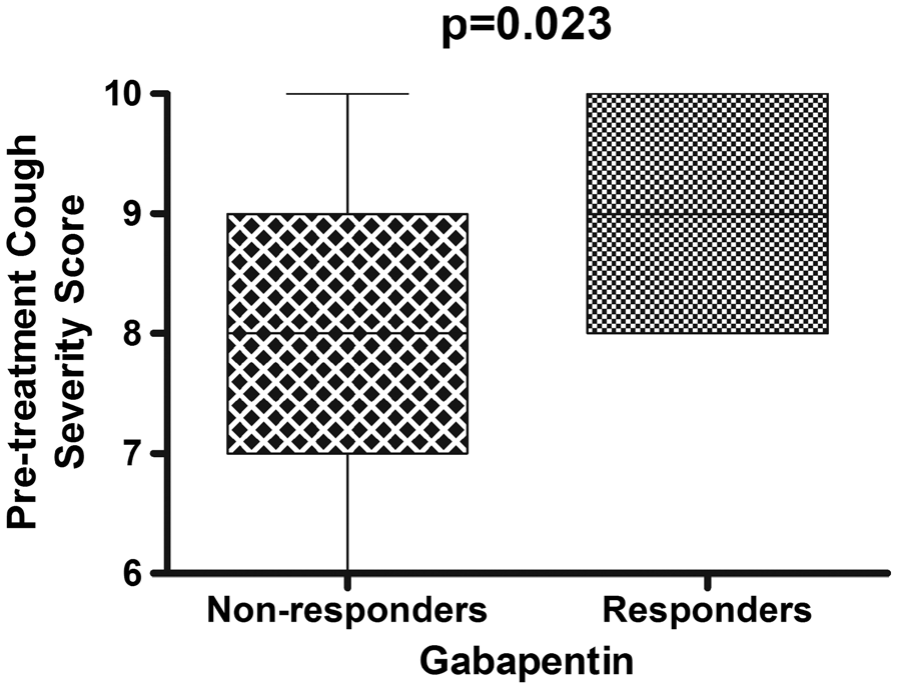
Comparison of cough severity score before gabapentin treatment between responders and non-responders: responders to gabapentin treatment showed significant higher pretreatment cough severity score (p=0.02).

We investigated if there were certain characteristics that predicted response to gabapentin. There was a significant difference in pre-treatment cough severity score between responders and non-responders. Responders have a higher subjective cough severity score before the treatment compared to non-responders (p=0.02) (Figure 2). Patients with a history of an upper airway infection also showed a significant higher improvement after gabapentin use than the other patients (p=0.04). There was no difference between responders and non-responders in terms of age, duration of cough, number of triggers, reflux or day/night predominance.

**Figure 3:**
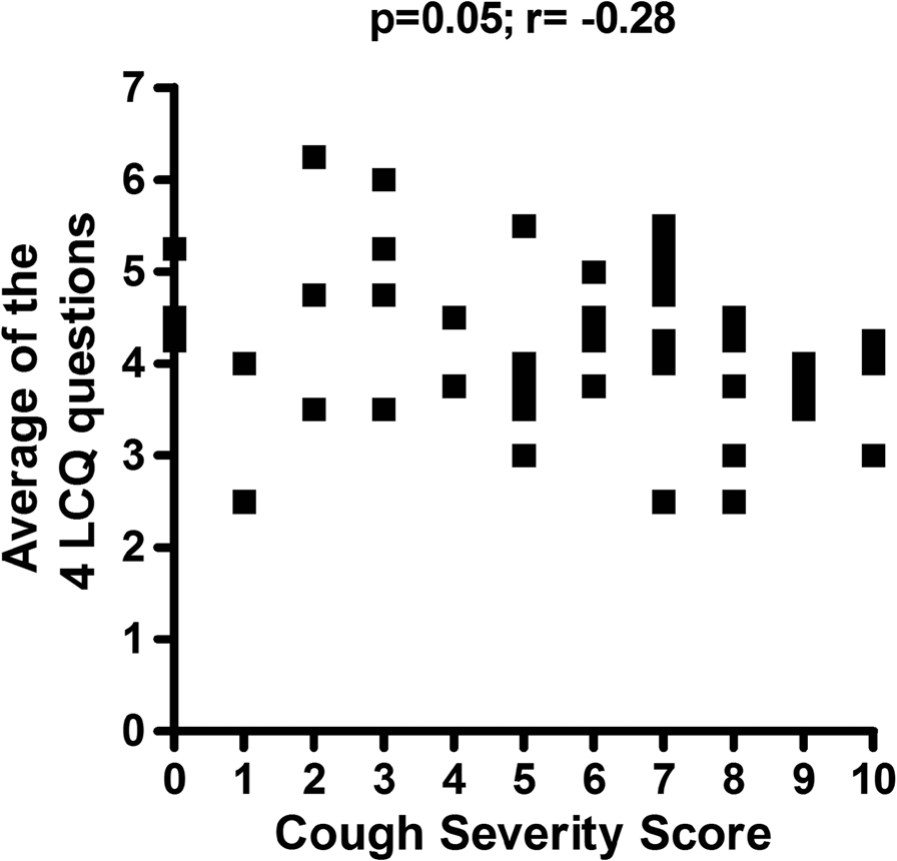
**Correlation between cough severity score and average of the four Leicester Cough Questionnaire scores: a significant negative correlation was seen between Leicester Cough Questionnaire and cough severity score (p=0.05; r= −0.28).** Lower scores in the Leicester Cough Questionnaire signify a higher impact of cough on daily life. LCQ = Leicester Cough Questionnaire.

Current cough severity score correlated with the average of the four Leicester Cough Questionnaire scores, indicating that cough severity score is a reliable tool to score cough severity (p=0.05; r= −0.28) (Figure 3).

## Discussion

Our results suggest that chronic idiopathic cough patients were predominantly middle-aged women, frequently reporting various cough triggers. Change in temperature, talking and strong odours are the most frequent triggers. We also show that gabapentin, 600 mg twice daily, might improve cough in patients with chronic idiopathic cough. Patients were more likely to respond to gabapentin if they had a history of an airway infection before the onset of the chronic cough and if they had a pre-treatment cough severity score higher than eight. The cough severity score, where patients score their cough severity on a scale of zero to ten, correlated with questions from the Leicester Cough Questionnaire.

Our patient characteristics are in line with the results from Haque et al. They reported that patients with idiopathic cough, often had an upper respiratory tract infection preceding their cough [11]. This was also seen in our patient cohort. We found that patients with chronic idiopathic cough were often middle-aged women with a long history of cough, confirming the distinct clinical phenotype suggested by Haque et al. and also found in the recent trial by Ryan and colleagues [8]. Patients clearly present with a longstanding cough problem. This is due to the fact that patients often have a long history of investigations and trial treatments before attending a tertiary outpatient clinic.

The improvement of cough severity with gabapentin treatment we see has previously been suggested in case reports [7] and in the recent randomised, double-blind, placebo-controlled trial by Ryan et al. [8]. The latter shows a mean improvement in cough severity of 11.1 mm while we saw a slightly higher improvement of 2.8 (which equals 28 mm). A similar rate of side effects was seen with fatigue and dizziness in 19%, but with a lower rate of nausea of 9% in our cohort. This might be due to the lower maximum dose of gabapentin we used (1200 mg vs 1800 mg) [8]. We found that a subset of patients did not benefit from gabapentin treatment. This reflects the heterogeneity of chronic idiopathic cough where refractory cough is caused by many disorders [12-15]. The analysis of the cough scores showed that it is particularly effective in a subgroup of patients with a high initial cough score and in patients who previously had an airway infection. Further studies are warranted to unravel the exact mechanism of action of gabapentin in chronic idiopathic cough.

The triggers found to be associated with cough in chronic idiopathic cough patients are more or less in line with literature where temperature changes, talking, eating and smoke or fragrances are described as the most prominent [16]. Patients that responded to the gabapentin therapy also mentioned that their cough response to these triggers was decreased. This is in contrast with the findings of Ryan et al. who could not show a significant change in peripheral cough reflex sensitivity, suggesting that gabapentin did not act by reducing peripheral sensitisation [8]. In our analysis, the decreased response to those triggers was a subjective feeling the patient had following the use of gabapentin, whereas Ryan et al. used an objective single-dose capsaicin cough reflex sensitivity method.

There are however limitations to our results. Patients were asked to recall severity of cough before onset of treatment and therefore recall bias might influence the results. We do not think that this will influence the results much as each patient clearly recalled whether gabapentin improved their cough. As this was not a placebo-controlled trial, the effect could also be attributed to a placebo effect. Nonetheless we have to bear in mind that these patients previously had multiple other treatments without any benefit. Subsequently, the majority of them were very reluctant in trying yet ‘another’ treatment. Our uncontrolled, open-label study results confirm the recent findings of Ryan and colleagues [8].

## Conclusion

In conclusion, we show that chronic idiopathic cough were predominantly middle-aged women and demonstrated that gabapentin can significantly improve cough in these patients. Responders tend to have higher pre-treatment severity scores and have a history of an airway infection. Further randomized, placebo controlled studies are warranted to confirm these findings and more research is needed to unravel the mechanisms by which gabapentin improves cough.

## Abbreviations

CT: Computed tomography; IQR: Interquartile range; SD: Standard deviation; LCQ: Leicester Cough Questionnaire.

## Competing interests

All authors agreed with the final draft of the manuscript and they have no competing interests.

## Authors’ contributions

CVdK gathered the data and wrote the first drafts of the article. PCG analyzed the data and wrote the final draft of the article. CVdK and PCG both contributed equally to the final manuscript. PVB and LJD both contributed to the concept of the study and critically revised the manuscript.

## Authors’ information

Van de Kerkhove C and Goeminne P.C are joint first authors.
